# Effects of Apatinib combined with Temozolomide on levels of sPD-1 and sPD-L1 in patients with drug-resistant recurrent glioblastoma

**DOI:** 10.1016/j.clinsp.2024.100376

**Published:** 2024-05-10

**Authors:** Ren Zhao Kuang, Jun Wang, Yuan Chuan Wang, Xiao Ping Tang

**Affiliations:** a Department of Neurosurgery, Affiliated Hospital of North Sichuan Medical College, Nanchong City, Sichuan Province, China; b Department of Orthopedics and Cosmetology, Affiliated Hospital of North Sichuan Medical College, Nanchong City, Sichuan Province, China

**Keywords:** Glioblastoma, Recurrence, Apatinib, Temozolomide, Soluble PD-1, Soluble PD-L1

## Abstract

**Objective:**

This study aimed to explore the effects of Apatinib combined with Temozolomide (TMZ) on the levels of Soluble PD-1 (sPD-1) and Soluble Programmed Death-1 Ligand (sPD-L1) in patients with drug-resistant recurrent Glioblastoma (GB).

**Study design:**

A total of 69 patients with recurrent GB from September 2020 to March 2022 were recruited and assigned to the control group (n = 34) and observation group (n = 35) according to different treatment options after tumor recurrence. The control group was treated with TMZ, and the observation group was treated with Apatinib combined with TMZ. Levels of sPD-1 and spd-l1, clinical efficacy, survival time and adverse reactions were observed and compared between the two groups.

**Results:**

General data including gender, age, body mass index, and combined diseases indicated no statistical significance between groups (p > 0.05). Before the intervention, sPD-1 and sPD-L1 levels were not significantly different in the two groups (p > 0.05). After interventions, levels of PD-1 and sPD-L1 levels decreased significantly (p < 0.05). The objective remission rate and clinical benefit rate of the observation group were higher and overall survival and progression-free survival were longer than those of the control group (p < 0.05). No significant difference was observed in major adverse reactions among patients (p > 0.05).

**Conclusions:**

Apatinib combined with TMZ is safe and effective in the treatment of recurrent GB. The combined application of the two can reduce the levels of sPD-1 and sPD-L1, which has important clinical application value.

## Highlights

• Apatinib combined with TMZ is effective for recurrent GB.
• Apatinib combined with TMZ can reduce the levels of sPD-1 and sPD-L1.

## Introduction

Glioblastoma (GB) is the most common malignant tumor of the central nervous system, showing invasive growth, rapid progression, short survival, and a high recurrence rate.^1^ In previous prospective clinical trials, patients with recurrent GB had a poor prognosis, with a median Overall Survival (OS) of only 30‒39 weeks.^2^ Recommendations for GB include surgical resection where possible, followed by concurrent intensive chemotherapy with Temozolomide (TMZ) or chemotherapy with cisplatin, carboplatin, cyclophosphamide, and irinotecan.^3^^,^^4^ However, the effect of such treatment is not ideal, tumor tolerance to chemoradiotherapy is poor, and conventional chemotherapy drugs are not easily pass the blood-brain barrier.^5^ Therefore, there is no standard and effective treatment strategy for relapsed and advanced GB patients once first-line treatment fails. Apatinib is of antitumor efficacy in tumors.^6^ At present, Apatinib has been gradually applied in the treatment of recurrent glioma, mostly in scattered reports. In this study, patients with recurrent GB were selected and divided into two groups according to different treatment regimens to compare the clinical efficacy and adverse reactions of Apatinib combined with TMZ, as well as its influence on sPD-1 and sPD-L1 levels in patients.

## General information and methods

### General information

This prospective cohort study was performed following the Strengthening the Reporting of Observational Studies in Epidemiology (STROBE) Statement. A total of 69 patients with recurrent GB who were admitted to the Affiliated Hospital of North Sichuan Medical College from September 2020 to March 2022 were recruited, aged 25 to 71 years old, with an average age of (48.67 ± 12.54) years old. They were assigned to the control group (n = 34) and the observation group (n = 35) according to different treatment options after tumor recurrence. The control group was treated with TMZ, and the observation group was treated with Apatinib combined with TMZ. All patients were informed of the treatment plan and possible complications in detail before treatment and signed chemotherapy treatment consent. This study has been approved by the ethics committee of the Affiliated Hospital of North Sichuan Medical College (nº 20206SC32).

### Criteria

#### Inclusion criteria

(1) Age > 18 years old; (2) All patients received conventional radiotherapy and synchronous TMZ 4 weeks after surgery; (3) Recurrence of grade Ⅲ or Ⅳ GB was confirmed by postoperative pathological and imaging data; (4) Postoperative MRI indicated total resection or subtotal resection of the tumor, and at least all T1 enhanced signals were removed in patients located in functional areas. (5) Karnofsky score ≥70 and expected survival ≥ 3 months.

#### Exclusion criteria

(1) Recent participation in other clinical trials or use of interfering drugs; (2) Intolerance to the drugs used in this study; (3) Patients with dysphagia, chronic diarrhea or intestinal obstruction that significantly affect oral drug absorption; (4) Only partial excision or biopsy was performed; (5) Patients with severe hepatic and renal insufficiency; (6) Patients with allergic constitution, autoimmune disease, blood system disease, malignant tumor; (7) Patients with incomplete laboratory examination; (8) Other situations that may affect clinical research and the judgment of research results.

## Methods

The control group was given oral TMZ (Jiangsu Tasly DIYI Pharmaceutical Co., Ltd., batch nº 20160083), 150 mg/(m^2^/d), every other week for 7 days (Days 1‒7, 15‒21), a cycle of 28 days.

The observation group was also given oral Apatinib Mesylate Tablets (Jiangsu Hengrui Pharmaceuticals Co., Ltd., batch number: H20140103) on the basis of TMZ, 500 mg each time, once a day. Drug therapy was continued until disease progression or the follow-up deadline, or drug therapy was discontinued due to intolerance.

Granisetron or metoclopramide was given accordingly to reduce digestive tract reactions. Mannitol, methylprednisolone sodium succinate, or dexamethasone were given to reduce edema around the tumor as shown in the imaging data. Symptomatic treatments were given to treat epilepsy and headache. Blood pressure, liver and kidney function, and blood and urine routine were monitored during treatment.

### Laboratory indicators

All patients were instructed to draw 5 mL of peripheral venous blood in the morning fasting state. Then the blood sample was placed in the anticoagulant tube, centrifuged at 3000 r/min for 10 min, and then the upper serum was absorbed and stored at -80°C. sPD-1 and sPD-L1 were detected by enzyme-linked immunosorbent assay. The standard product was prepared, and the sample was diluted (1:2). Serum to be tested and standard substance were added at 100 μL/well successively, and the enzyme label plate was coated with film and incubated at 37°C for 2h. The reaction plate was cleaned 3 times, added with a biotinized human ANG antibody working solution at 100 μL/ well, and incubated at 37°C for 30 min. The reaction plate was cleaned for 4 times, and ABC working solution was added into the reaction well at 100 μL/well and wet-incubated at 37°C for 45 min. After color development, the plate was incubated for 20 min away from light and added with a termination solution at 100 μL/well. Absorbance values were measured at 450 nm, and the color response depth was proportional to the level of relevant indicators, which was calculated by drawing a standard curve.

### Adverse events

(1) Hand and foot syndrome: Oral drug treatment could be continued in patients with grade 1 hand and foot syndrome, drug dose could be appropriately reduced for patients with grade 2 hand and foot syndrome, and drug treatment should be suspended for grade 3. Urea ointment or magnesium sulfate treatment may be given.
(2) Albuminuria: Grade 1 (albuminuria+ or 24h urinary protein < 1.0g) and Grade 2 (albuminuria++ or 24h urinary protein 1.0‒3.4g) can continue to use drugs, without reduction. Grade 3 (urinary protein+++ or 24h urinary protein > 3.5g) should be stopped drug treatment; If parallel treatment can restore albuminuria to below grade 2, drug treatment can be continued after dose adjustment; Treatment should be terminated if grade 3 albuminuria still occurs after 2 dose reductions.
(3) Hypertension: Adverse hypertensive reactions were treated with antihypertensive drugs orally. If grade 3 hypertension occurs, the drugs should be stopped, and after blood pressure control, drug treatment can be continued. If grade 4 hypertension (malignant hypertension, hypertensive crisis) occurs, the treatment should be terminated.
(4) Bleeding: If massive gastrointestinal bleeding occurs, treatment should be terminated immediately.

### Curative effect criteria

MRI was performed after 1 month of treatment to measure the maximum tumor diameter, transverse diameter, and coronial height in an enhanced axial image. According to the Flair image, the maximum diameter of peritumor edema before and after 1 month of treatment was observed to calculate tumor size (cm^3^) and edema degree. According to image data within 1 month, Response Evaluation Criteria in Solid Tumors (RECIST) were applied to evaluate the tumors. Complete Relief (CR): all tumor lesions disappeared and remained for more than 4 weeks; Partial Relief (PR): the total diameter of all tumor lesions was ≥30% lower than baseline; Progression Disease (PD): Tumor lesions increased in diameter and relative by at least 20%, or one or more new lesions appeared; Stable Disease (SD): The reduction degree of tumor lesions did not reach PR, and the increase degree did not reach PD level. Objective Response Rate (ORR) = (CR + PR)/(CR + PR + SD + PD) ×100%; Disease Control Rate (DCR) = (CR + PR + SD)/(CR + PR + SD + PD) ×100%.

### Survival time

All patients were followed up until death or expiration date. Overall Survival (OS) and Progression-Free Survival (PFS) were calculated from the start of treatment to the date of death or to disease progression. Kaplan-Meier method calculated the percentage of 6-month PFS (the percentage of patients without disease progression or death within 6 months).

### Termination of treatment and withdrawal from study

Medical imaging (RANO standard) findings of disease progression or recurrence, severe adverse reactions to Apatinib, and inability to tolerate treatment despite dose reduction are not required to give a reason and can be withdrawn from the study at any time.

### Statistical analysis

SPSS 24.0 statistical software was used to analyze the data. Measurement data conforming to normal distribution were expressed as mean ± standard deviation (x̄±s), and a t-test was used for comparison between groups. Enumeration data were expressed by the number of cases (n) or percentage (%) and compared by the Chi-Square test. Kaplan-Meier method calculated the survival rate, and the Log-Rank test was used for survival analysis and comparison; p < 0.05 represented a significant difference.

## Results

### General information

Table 1 shows no statistically significant difference in general data including gender, age, body mass index, and combined diseases in the two groups (p > 0.05).

**Table 1:** Comparison of general data between the two groups ($\bar{x}$ ± s, %).

Groups			Control group (n = 34)	Observation group (n = 35)	Statistical value	p
Gender	Male		24	27	2.541	0.057
	Female		10	8		
Age (years)			48.66 ± 12.01	48.63 ± 12.73	1.007	0.133
Mean recurrence time (months)			8.12 ± 3.17	8.48 ± 3.22	1.276	0.091
Tumor site		Solitary	26	29	0.765	0.298
		Multifocal	8	6		

### sPD-1 and sPD-L1 levels

Neither sPD-1 nor sPD-L1 indicated significance between the 2 groups before intervention (p > 0.05). Combined treatment lowered sPD-1 and sPD-L1 in a more effective manner (p < 0.05) (Table 2).

**Table 2:** Comparison of laboratory indexes between the two groups ($\bar{x}$ ± s).

Groups		Control group (n = 34)	Observation group (n = 35)	*t*	p
sPD-1	Before intervention	17.34 ± 2.43	17.35 ± 2.41	1.063	0.712
	After intervention	14.71 ± 1.24	6.37 ± 0.93	8.137	0.002
sPD-L1	Before intervention	19.04 ± 3.46	19.05 ± 3.41	0.927	1.82
	After intervention	16.71 ± 2.43	11.37 ± 1.35	8.575	0.002

### Clinical efficacy

Combined treatment increased ORR and CBR in patients compared with single TMZ treatment (p < 0.05) (Table 3).

**Table 3:** Comparison of clinical efficacy between the two groups ($\bar{x}$ ± s).

Groups	Control group (n = 34)	Observation group (n = 35)	*t*	p
CR	0 (0.00)	3 (8.5)	‒	‒
PR	8 (23.53)	16 (45.71)		
SD	12 (35.29)	11 (37.14)		
PD	14 (41.18)	5 (14.28)		
ORR	8 (23.53)	19 (54.28)	8.421	0.002
CBR	20 (58.82)	30 (85.71)	10.217	0

### Survival

OS and PFS were longer in patients after combined treatment compared with TMZ treatment (p < 0.05) (Table 4).

**Table 4:** Comparison of blood lipid difference before and after treatment between the two groups ($\bar{x}$ ± s).

Groups	Control group (n = 34)	Observation group (n = 35)	*t*	p
Overall survival (months)	8.73 ± 5.57	14.27 ± 4.77	10.596	0
Progression-free survival (months)	5.27 ± 3.79	11.87 ± 4.78	10.849	0

### Adverse reactions

Major adverse reactions among patients suggested no statistically significant difference (p > 0.05) (Table 5).

**Table 5:** Comparison of adverse reactions between the two groups (%).

Groups	Control group (n = 34)	Observation group (n = 35)	X^2^	p
Elevated blood blood pressure (grade 1)	2 (5.88)	3 (8.57)	0.593	0.294
Nausea and vomiting (grade 1)	3 (8.82)	2 (5.71)		
Albuminuria (grade 1)	0 (0.00)	1 (2.85)		
Myelosuppression (grade 1)	1 (2.94)	0 (0.00)		
Hand-foot syndrome (grade 1)	1 (2.94)	1 (2.85)		

## Discussion

GB takes on characteristics of rapid progression, low survival rate, and extremely poor prognosis.^7^ At present, although with standard treatments, the clinical efficacy is poor. For recurrent GB cases, there is still no standard treatment for patients who refuse to undergo re-surgery or are not candidates for re-surgery. Postoperative chemoradiotherapy or molecular targeted therapy are the main therapeutic methods, both of which aim to reduce and destroy tumor cells and inhibit tumor cell proliferation. However, due to drug resistance, therapeutic efficacy has been at a bottleneck, which is mainly related to ignoring the host immune system in malignant tumors.^8^ TMZ intensive therapy is a commonly used therapy regimen. However, TMZ therapy is MGMT promoter methylation-dependent with limited long-term efficacy. Apatinib is a small-molecule tyrosine kinase inhibitor independently developed in China, which can highly selectively compete with intracellular binding sites, block downstream cell signals, and thus inhibit angiogenesis in tumor tissues.^9^^,^^10^ Apatinib has ideal efficacy in gastric cancer, esophageal cancer, lung cancer, liver cancer, colorectal cancer, etc.^11^ sPD-1 is mainly expressed on the surface of activated T-cells, B-cells, and macrophages, and its ligands include sPD-L1 and s PD-L2. As a transmembrane transport glycoprotein, sPD-L1 is an immunosuppressive molecule in the human body and widely exists in tumor cells such as lymphocytes.^12^ Changes in sPD-L1 levels can reflect neovascularization and apoptosis. When the sPD-L1 level increases in the body, it can bind more to the sPD-1 receptor, inhibit T-cell proliferation and differentiation and induce apoptosis, suppressing the normal immune system, making malignant proliferating tumor cells escape immune killing, resulting in the obstruction of the apoptosis process of cancer. During carcinogenesis, driving carcinogenic events may lead to overexpression of PD-L1. For example, Epidermal Growth Factor Receptor (EGFR) mutations are positively correlated with the expression of PD-L1 in lung cancer, and EGFR inhibitors can inhibit the transcription of PD-L1.^13^ Clinical studies have found that radiotherapy can induce sPD-L1 in tumor cells, and sPD-L1 promotes tumor immune escape.^14^, ^15^, ^16^ In recent years, tumor immunotherapy for PD-1 or PD-L1 has been proven to cause durable anti-tumor immune response in many types of tumors with less toxicity. Although there is still much to be learned about this signaling pathway, PD-1/PD-L1 blocking therapy is still expected to become the main immunotherapy for cancer in the next few years.^17^, ^18^ This study observed that sPD-1 and sPD-L1 levels were effectively reduced after combined treatment. Analysis suggested that Apatinib combined with TMZ specifically binds to the competitive target cells of sPD-1 and sPD-L1 to activate the target cell membrane, thus reducing and weakening the immune effect.^19^^,^^20^

As the second-generation alkylating agent, TMZ can directly pass the blood-brain barrier, alkylating the 6^th^ oxygen atom of guanine on DNA molecules, and play a cytotoxic role through the misallocation repair of methylated admixtures. It is currently a first-line chemotherapy drug for glioma.^21^ Although TMZ has achieved certain clinical efficacy, glioma is prone to develop resistance to TMZ. The dose density scheme is to regenerate the cytotoxic effects of TMZ in drug-resistant glioma by depleting MGMT activity in blood monocytes at a continuous low dose.^22^ Apatinib has shown significant improvement in cancer patients’ outcomes, as well as in recurrent GB patients who did not respond to bevacizumab.^23^ This study showed that combined treatment reduced peritumoral edema and shrunk tumor size, as well as increased clinical ORR, CBR, OS, and PFS. These results indicated that the treatment effect of Apatinib combined with TMZ intensive regimen was significantly better than that of TMZ alone, and Apatinib could enhance the antitumor effect of TMZ or partially reverse TMZ resistance in patients with recurrent GB. Analysis suggested that Apatinib combined with TMZ could significantly reduce sPD-L1 in patients, reduce its binding to sPD-1 receptor, promote T cell activation, and avoid cancer cells from escaping the anti-tumor immunity of the body.^24^ Apatinib can promote glioma cell apoptosis and inhibit cell proliferation, and the combination with TMZ further reduces cell proliferation and improves antitumor activity.^25^^,^^26^ In terms of adverse reactions, patients mainly suffered from elevated blood pressure, digestive tract reaction, myelosuppression, hand and foot rash, pruritus, desquamate, rhagades, and transient proteinuria during medication. The reactions were mild, mainly grade 1‒2, and were quickly controlled and improved after symptomatic treatment, indicating that Apatinib combined with TMZ had higher safety.

In conclusion, Apatinib combined with TMZ improved the symptoms of patients with recurrent GB in the short term, alleviated clinical symptoms, and reduced sPD-1 and sPD-L1 in peripheral blood. However, the results may be biased because of the small case size and short follow-up time, and larger samples and multi-center studies are needed to further observe the long-term efficacy of this program.

### Ethical statement

All procedures performed in this study involving human participants were in accordance with the ethical standards of the institutional and/or national research committee and with the 1964 Helsinki Declaration and its later amendments or comparable ethical standards. All subjects were approved by the Affiliated Hospital of North Sichuan Medical College (nº 20206SC32).

### Author's contribution

R. Kuang designed the research study. J. Wang and Y. Wang performed the research. X. Tang and Y. Wang provided help and advice. R. Kuang and J. Wang analyzed the data. R. Kuang wrote the manuscript. X. Tang reviewed and edited the manuscript. All authors contributed to editorial changes in the manuscript. All authors read and approved the final manuscript.

## Funding

Nanchong City 2022 City School Science and Technology Strategic Cooperation Special Fund (Fund number: 22SXQT0039).

## Conflicts of interest

The authors declare no conflicts of interest.
